# Intra-Aortic Balloon Pump Rupture and Entrapment

**DOI:** 10.1155/2014/378672

**Published:** 2014-01-23

**Authors:** Artan Jahollari, Atilla Sarac, Ertugrul Ozal

**Affiliations:** Department of Cardiovascular Surgery, Medical Park Hospital, 55200 Samsun, Turkey

## Abstract

Intra-aortic balloon pump is used frequently to support a failing myocardium in cardiac patients. Due to the invasive nature of this device, usage is accompanied by consistent risk of complications. Balloon rupture, although it occurs rarely, may lead to entrapment if diagnosis delays. A 78-year male who underwent cardiac surgery experienced balloon rupture and entrapment in the right femoral artery during the postoperative follow-up. Surgical extraction under local anesthesia was performed and the patient had an uneventful course. Fast and gentle solution of the problem is necessary to prevent further morbidity or mortality related to a retained balloon catheter in these delicate patients.

## 1. Introduction

Intra-aortic balloon pump (IABP) counterpulsation is a very helpful tool in management of critic cardiac patients, both in cardiology and cardiovascular surgery units. It was first introduced by Kantrowitz et al. in 1967, and ever since its indications have significantly increased [1]. During decades, catheters have become easy to insert or extract, and the device itself can be simply controlled by even nonsenior health workers.

However, despite all the technological progress that has been made, there is still a high rate of complications regarding IABP usage. Most of them include vascular complications such as limb ischemia, thromboembolism, visceral ischemia, and spinal cord injury [1]. Balloon rupture and entrapment are a rarely seen complication. There is limited literature regarding this topic, mostly in the form of case reports. We present a case where the balloon ruptured and was entrapped in femoral artery during extraction, which was solved surgically.

## 2. Case Report

A 78-year old male, who had had a previously left internal mammarian artery (LIMA) and left anterior descending artery (LAD) bypass before 10 years, presented with angina pectoris and resting dyspnea. He also suffered from diabetes, obesity, and chronic obstructive lung disease. Echocardiography revealed dilated left heart chambers, ejection fraction of 30%, mild mitral regurgitation, and elevated pulmonary pressure. Coronary artery angiography demonstrated 80% stenosis of left main coronary artery, severe lesions of LAD and circumflex artery (Cx), total occlusion of LIMA, and noncritical lesions of right coronary artery. The patient underwent a double vessel off-pump coronary artery bypass grafting with saphenous grafts to LAD and Cx.

Due to significant symptomatic status and poor coronary artery quality, preoperative IABP of 7.5 F and 40 cc size was inserted. Catheter was inserted percutaneously and sheathless via the right femoral artery, without encountering any obstacles. Counterpulsation was arranged at 1 : 2 ratio. In the follow-up the patient was extubated and had stable hemodynamic parameters.

**Figure 1:**
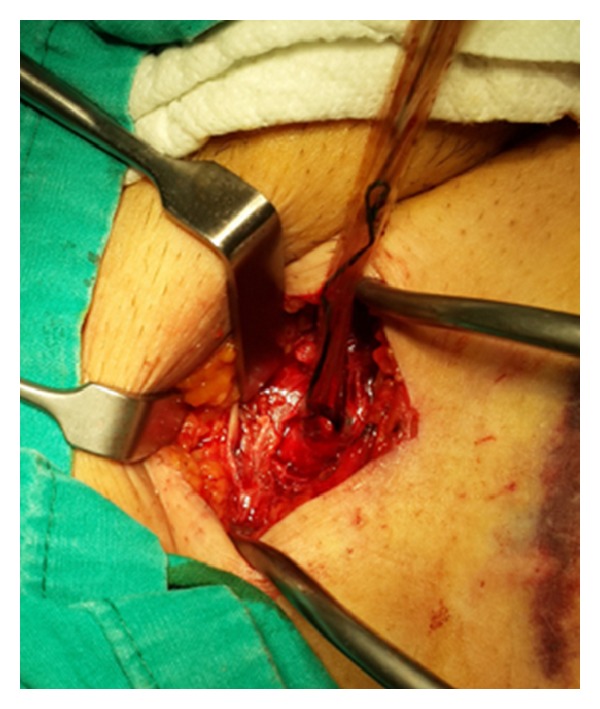
View of the retained balloon catheter.

**Figure 2:**
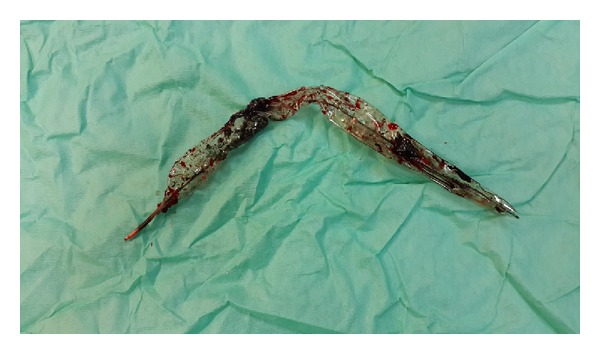
Firm thrombus inside the balloon.

18 hours later IABP discontinued to work because of leakage. Blood appearance at both safety chamber and helium catheter was noticed. IABP was immediately stopped and resistance was encountered during extraction. Gentle traction was applied and the catheter was half-way extracted, while hard blood clots were noticed inside the balloon. Insertion of a larger sheath and thrombolytic injection were tried without success. At the point when only the tip of balloon remained entrapped (Figure 1), it was realized that it was impossible to retain it without leading to a large damage of the vessel. Under local anaesthesia, punction site at the femoral artery was localized through a small skin incision, artery was clamped, and arteriotomy was extended. After extracting the catheter (Figure 2), femoral artery was repaired primarily and the patient had an eventful postoperative course.

## 3. Discussion

IABP has been used for decades as an advanced treatment modality in myocardial infarction and its complications, low cardiac output syndrome after cardiac surgery, bridge to transplantation, and so forth. Based on simple mechanic principles it augments coronary perfusion and decreases left ventricle afterload, supporting the physiology in a failing myocardium. During the years, different and more sophisticated catheters and devices have been presented, leading to faster and easier management.

However, complication rate related to IABP usage remains still significant. Extremity ischemic sequels comprise 87% of complications. It occur as often as 8–18%, while major complications compromising the limb are less than 1% [2]. Female gender, diabetes, and peripheral artery disease are predisposing factors. Also, sheathed insertion is suggested as a modifiable factor. Perforation of the balloon is a rare adverse event occurring in less than 0.5%. Abrasion of the balloon by a heavily calcified aortic wall is kept responsible for the rupture. It was first reported by Rajani, followed by Milgalter and Aru [3–5]. Blood that enters the perforated balloon is entrapped due to the negative pressure of deflation and reacts rapidly with helium to form solid clots. Leakage alarm and loss of augmentation followed by blood appearance in the safety chamber points to IABP perforation and prompt extraction are mandatory. In our case, probably due to overlook, there might have been some delay until diagnosis of the problem.

Percutaneous withdrawal using larger sheathes or thrombolytic injection in the balloon has been suggested [6, 7]. In our case thrombolytic injections were not an option, as the balloon and the catheter were damaged in different points during extraction, and injected agents could not reach the entrapped part. First, we used a 9 F sheath to retain the catheter. The attempt was unsuccessful as the thrombus was large and firm. Then, we decided for surgical excision under local anesthesia, as delay of the problem might lead to further morbidity.

Although easy and comfortable to use, IABP necessitates a vigilant follow up due to a significant complication rate. When rupture of the balloon is noticed, it should be promptly extracted, to avoid entrapment due to rapid formation of solid thrombus. Surgery is advisable for hard-to-pull entrapped catheters, violent extraction of which may lead to major arterial injury.

## References

[1] Kantrowitz A, Wasfie T, Freed PS (1986). Intraaortic balloon pumping 1967 through 1982: analysis of complications in 733 patients. *American Journal of Cardiology*.

[2] Meharwal ZS, Trehan N (2002). Vascular complications of intra-aortic balloon insertion in patients undergoing coronary reavscularization: analysis of 911 cases. *European Journal of Cardio-Thoracic Surgery*.

[3] Rajani R, Keon WJ, Bedard P (1980). Rupture of an intra-aortic balloon. A case report. *Journal of Thoracic and Cardiovascular Surgery*.

[4] Milgalter E, Mosseri M, Uretzky G, Romanoff H (1986). Intraaortic balloon entrapment: a complication of balloon perforation. *Annals of Thoracic Surgery*.

[5] Aru GM, King JT, Hovaguimian H (1986). The entrapped balloon: report of a possibly serious complication. *Journal of Thoracic and Cardiovascular Surgery*.

[6] Brodell GK, Tuzcu EM, Weiss SJ, Simpfendorfer C (1989). Intra-aortic balloon-pump rupture and entrapment. *Cleveland Clinic Journal of Medicine*.

[7] Fitzmaurice GJ, Collins A, Parissis H (2012). Management of intra-aortic balloon pump entrapment: a case report and review of the literature. *Texas Heart Institute Journal *.

